# Postoperative lingual nerve injury following airway management: A literature review

**DOI:** 10.1177/17504589241270238

**Published:** 2024-08-27

**Authors:** Mohamed Aly, Rohan Dadak, Cheng Lin, Kamal Kumar

**Affiliations:** 1 Schulich School of Medicine and Dentistry, The University of Western Ontario, London, ON, Canada; 2 Michael G. DeGroote School of Medicine, McMaster University, Hamilton, ON, Canada; 3 London Health Sciences Centre, London, ON, Canada

**Keywords:** Intubation, Lingual nerve neuropraxia, Anaesthesia, Laryngeal mask airway

## Abstract

Postoperative lingual nerve injury is a rare but serious complication following airway management and can lead to significant discomfort and disability. This literature review explores the aetiology, clinical presentation, management strategies and potential preventive measures for lingual nerve injuries associated with airway management during surgery. A search of PubMed, MEDLINE, EMBASE Science Direct, Cochrane library and Web of Science databases was done since inception to January 2024, including any observational studies and clinical trials describing patients diagnosed with lingual nerve injury following airway instrumentation. Multiple risk factors for lingual nerve injury were identified. Anaesthesia factors include difficulty with intubation and use of laryngeal mask airway. Surgical factors are long duration of operation and surgery of the head and neck. Patient factor includes female sex. Anaesthetists should proactively inform patients about the potential for this nerve injury and control modifiable risk factors to mitigate the risk of injury.

## Key Phrases

- Lingual nerve injury is a complication following airway management during surgery.
- Lingual nerve injury commonly presents as tongue numbness and altered taste.
- Many risk factors for lingual nerve injury include the sex of the patient, airway device, type of surgery and length of surgery.
- Management of lingual nerve injury includes expectant management, medical management using anti-inflammatories and multidisciplinary management of long-term sequelae.

## Introduction

The lingual nerve is the terminal branch of the mandibular division of the fifth cranial nerve. It provides taste sensation to the front two-thirds of the tongue and innervates the submandibular and sublingual glands (Fagan & Roy 2023). It is located under the roots of the third molar and on the inner surface of the mandible, making it vulnerable to injury (Laxton & Kipling 1996).

Lingual nerve neuropraxia commonly presents as tongue numbness and altered taste in the anterior two-thirds of the tongue (Su et al 2018). Due to its proximity to other cranial nerves, a lingual nerve injury may not be isolated and may present with hypoglossal, glossopharyngeal, or recurrent laryngeal nerve damage.

Airway management can also lead to lingual nerve injury, with factors such as inappropriate-sized laryngeal mask airway (LMA), multiple attempts, high pressure in laryngeal mask cuff and difficult intubation (Brimacombe et al 2005). High pressure in the supraglottic airway devices may compress or stretch the nerve at the tongue’s edge or the mandible’s inner surface near the third molar and causes nerve injury (Thiruvenkatarajan et al 2015).

In addition, numerous medical conditions have been considered as risk factors, such as ankylosing spondylitis, rheumatoid arthritis and calcinosis, Raynaud phenomenon, esophageal dysmotility, sclerodactyly and telangiectasia (CREST) syndrome (Brimacombe et al 2005).

Limited literature exists on its incidence, risk factors, presentation and treatment beyond individual case reports. This review aims to consolidate available literature and compile evidence on the management of lingual nerve injury.

### Search strategy

**Figure 1:**
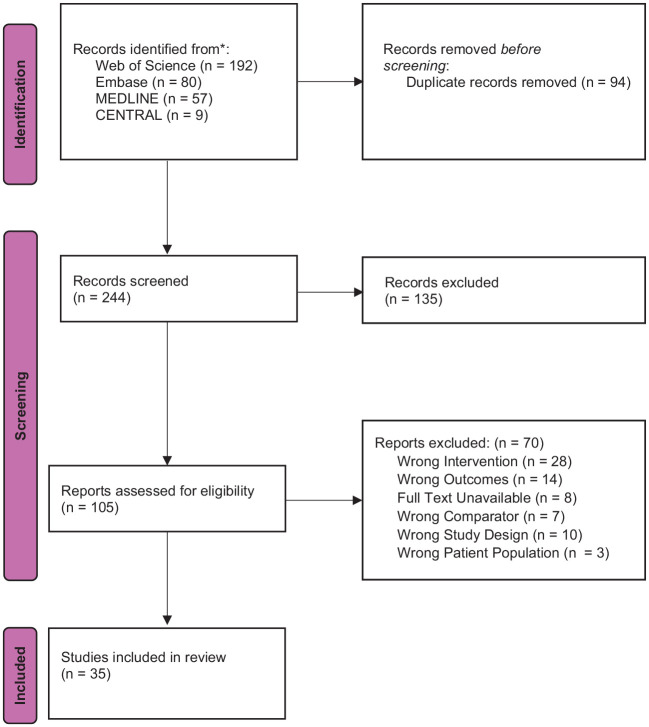
PRISMA flowchart summarising the process of article selection

We conducted a review of the literature to evaluate the current trends in postoperative tongue numbness following airway management. The Preferred Reporting Items for Systematic Reviews and Meta-Analysis (PRISMA) checklist is detailed in Figure 1.

A search of MEDLINE, EMBASE, Cochrane Library and Web of Science databases was performed for all literature until January 2024. The search terms were curated to identify literature discussing lingual nerve damage following airway management by intubation or laryngoscopy for surgical intervention. Detailed search terms to retrieve eligible studies were attached in an Appendix.

### Inclusion criteria

We included literature discussing lingual nerve injury following airway management during surgical procedures. Observational studies, cohort studies, case reports, randomised control trials, clinical trials and multicentre studies were included. There were no restrictions placed on the publication year or geographical settings.

### Exclusion criteria

Exclusion criteria included letters to editors, conference abstracts and commentaries. Any studies that were not presented in English or lacked available full text were excluded. We also excluded review articles, systematic reviews and meta-analyses.

### Study selection

All the compiled literature from the databases was imported into Covidence. Automatic duplication checks were conducted by Covidence. Selected articles were screened by title and abstract, followed by a full-text assessment to ensure that these articles did indeed meet the eligibility criteria. Article selection was carried out by three independent reviewers with disagreements being resolved through a consensus decision. The PRISMA flowchart displays the number of studies included and excluded at each screening step (Figure 1).

### Data extraction

We extracted patient-related perioperative variables, such as age, sex, American Society of Anaesthesiologists (ASA) score, Mallampati (MP) score and comorbid diagnoses. We also extracted the type of airway management done, such as the use of endotracheal tube, LMA, cuffed oropharyngeal airway or laryngoscopy. In addition, the difficulty of intubation and size of the airway was noted. We extracted data regarding postoperative outcomes, such as presentation, time to onset and resolution of symptoms, investigations and treatments.

## Results

After removing duplicates, our search yielded 244 articles to screen. Following title and abstract screening, we had 105 articles available for full-text literature. After eliminating 70 studies, we included 35 studies in our review.

Our analysis identified 30 case reports that reported lingual nerve injury following airway management for 32 patients. Out of these, 21 patients had an LMA placed and 9 had an Endotracheal Tube (ETT) and 2 had Cuffed Oropharyngeal Airway (COPA)(Table 1).

**Table 1:** Summary of selected case reports along with patient demographics (age, gender, weight, MP score and American Society of Anesthesiologists (ASA) score), number of patients in each report, type of surgery, airway device used and site of tongue numbness

Author	Study design	Age and gender	Weight (kg)	No. of patients	Surgery	Airway	MP score	Site of tongue numbness	ASA
Afifi and Cozma 2023	Case report	62M	73	1	Laparoscopic cholecystectomy	Igel-4	1	Right side	2
Khashan et al 2022	Case report	52F	70	1	TAH	ETT	1	Left tongue numbness and taste	2
Li et al 2022	Case report	57F	65	1	Brachial plexus exploration	LMA-4	1	Anterior two-thirds of the tongue	1
Park and Jeong 2022	Case report	40F	41	1	Breast surgery	LMA-3	1	Right side	1
Wang et al 2021	Case report	49M		1	PCNL	LMA-4	1	Tip of the tongue numb	1
Kim et al 2018	Case report	61F		1	ORIF radius	ETT-7 plus airway	1	Anterior two-thirds	1
Ueshima et al 2016	Case report	53M	78	1	Total knee replacement	Igel-4	1	Bilateral tongue numbness	1
Jenkinson et al 2014	Case report	64F		1	Closed reduction elbow	Igel-4	1	Left tongue numbness	1
Thiruvenkatarajan et al 2014	Case report	45F	61	1	Tendon repair hand	LMA-supreme-3	1	Tip of the tongue	1
Ulusoy et al 2014	Case report	19F		1	Septoplasty	ETT 7.5mm	1	Two-thirds of the left tongue	1
Dhillon and O’Leary 2012	Case report	52F	65	1	EBUS	LMA-4	1	Anterior two-thirds	2
El Toukhy and Tweedie 2012	Case report	36F		1	Myomectomy	LMA-3	1	Anterior two-thirds	1
Rujirojindakul et al 2012	Case report	43F 33F	65 53	2	L5–S1 discectomy ovum pick up	LMA-4 Igel-3	1	Tip of the tongue numbness	1
Renes et al 2011	Case report	69M	78	1	Inguinal hernia repair	Igel-4	1	Bilateral tongue numbness	1
Foley et al 2010	Case report	50F 21M	101 79	2	Sling procedure Groin repair	ETT 7.5 LMA-5	2 1	Tip of the tongue. Anterior one-third	2 1
Cardoso et al 2007	Case report	36F	60	1	Removal of breast implant	LMA-3	1	Anterior two-third	1
Brimacombe and Keller 2005	Case report	64F	76	1	Hand surgery	LMA-4 ProSeal	1	Entire tongue numbness	1
Laffon et al 2001	Case report	32F	65	1	D&C	COPA	1	Bilateral causing entire tongue numb	1
Kadry and Popat 2001	Case report	29F	60	1	Left ankle arthroscopy	COPA	1	Left tongue numbness	1
Gaut and Williams 2000	Case report	37M	105	1	Excision of a right VC cyst	ETT 6.5mm	1	Right anterolateral	1
Evers 1999	Case report	56M	85	1	Transsphenoidal hypophysectomy	ETT 9mm	2	Left side with speech problem	2
Majumder and Hopkins 1998	Case report	27F		1	Arthroscopy left wrist	LMA-3	1	Entire tongue numbness	1
Laxton and Kipling 1996	Case report	42F	54	1	Diagnostic laparoscopy	LMA-3	1	Left anterolateral	1
Ahmad and Yentis 1996	Case report	26M	-	1	Unilateral varicose vein surgery	LMA-4		Right side	1
Silva et al 1992	Case report	57F		1	Hernia repair	ETT Multiple attempts	1	Bilateral causing entire tongue numb	1
Loughman 1983	Case report	39F	51	1	Breast surgery	ETT 7mm	1	Right side	1
Teichner 1971	Case report	44M		1	Excision of granuloma left lung base	ETT, difficult intubation	2	Right anterolateral	2

### Presentation of injury

Patients predominantly reported symptoms of loss of sensation in the anterior two-thirds of the tongue or taste disturbances either hours after surgery or at most 24 hours after the operation (Table 2). Patients may also suffer from an unpleasant taste of metal, old cheese, or ammonia. At times, they may encounter difficulty with word pronunciation (Hillerup 2007).

**Table 2:** Summary of case reports selected along with surgical factors (type of surgery, duration, patient position), site of tongue numbness, onset and duration of symptoms, and interventions used

Author	Surgery	Duration (minutes)	Intraop- position	Site of tongue numbness	Onset of symptoms	Intervention	Recovery
Afifi and Cozma 2023	Laparoscopic cholecystectomy	90	Supine	Right side	Immediate once awake	Observation	4 weeks
Khashan et al 2022	TAH		Supine	Left tongue numbness and taste	Immediate once fully awake	Observation	14 weeks
Li et al 2022	Brachial plexus exploration		Supine	Anterior two-thirds of the tongue	Immediate once fully awake	IV dexamethasone × 3 days	6 weeks
Wang et al 2021	PCNL	80	Prone	Numbness at the tip	Immediate once fully awake	Methylcobalamin and vitamin B1	3 weeks
Kim et al 2018	ORIF radius	170	Supine	Bilateral, anterior two-thirds	30 min postop	IV dexamethasone 10mg × 3 days	3 days
Ueshima et al 2016	Total knee replacement		Supine	Bilateral tongue numbness	Immediate once fully awake	Observation	2 weeks
Jenkinson et al 2014	Closed reduction elbow	300	Supine	Left side tongue numbness	Immediate once fully awake	Observation	6 weeks
Thiruvenkatarajan et al 2014	Tendon repair hand	105	Supine	Tip of the tongue	30 min postop	Observation	3 weeks
Ulusoy et al 2014	Septoplasty	180	Supine	Two-thirds of the left tongue	Second postop	Steroids, Vit. E, B1 and B6	12 weeks
Dhillon and O’Leary 2012	EBUS		Supine	Anterior two-thirds	Immediate once awake	Observation	4 weeks
El Toukhy and Tweedie 2012	Myomectomy	180	Supine	Anterior two-thirds	Immediate once awake	Observation	6 weeks
Rujirojindakul et al 2012	L5–S1 discectomy Ovum pick up	75 45	Prone Lithotomy	Tip of the tongue numbness	Next day morning	Observation	2 weeks 2 weeks
Renes et al 2011	Inguinal hernia repair	45	Supine	Bilateral tongue numbness	Immediate once fully awake	Observation	8 weeks
Foley et al 2010	Sling procedure Groin repair.	70 45	Supine Supine	Right side	Immediate once awake	Observation Observation	4 weeks 4 weeks
Cardoso et al 2007	Removal of breast implant	120	Supine	Anterior two-third	Immediate once awake	Observation	3 weeks
Brimacombe et al 2005	Shoulder replacement	150	Sitting	Left side	Immediate once awake	Observation	2 weeks
Brimacombe and Keller 2005	Hand surgery	120	Supine	Entire tongue numbness	Immediate once fully awake	Observation	12 hours
Laffon et al 2001	D&C	20	Lithotomy	Bilateral causing entire tongue numb	Immediate once awake	Observation	2hours
Lang and Waite 2001	Left knee arthroscopy	105	Supine	Bilateral tongue		Oral steroids	19 months
Kadry and Popat 2001	Left ankle arthroscopy	65	Supine	Left tongue numbness	Immediate once fully awake	Observation	10 days
Gaut and Williams 2000	Excision of a right VC cyst	45	Supine	Anterolateral plus, loss of taste sensation	Postop	Observation	3 weeks for numbness and 12 weeks taste sensation
Evers 1999	Transsphenoidal hypophysectomy	180	Supine	Left side	Postop	Observation	8 weeks
Majumder and Hopkins 1998	Arthroscopy left wrist	20	Supine	Entire tongue numbness	Immediate once awake	Observation	6 weeks
Laxton and Kipling 1996	Diagnostic laparoscopy	35	Supine	Left anterolateral	Immediate once awake	Observation	16 weeks
Ahmad and Yentis 1996	Unilateral varicose vein surgery	30	Supine	Right side	Immediate once awake	Observation	—
Brimacombe 1993	ORIF radius	120	Supine	Bilateral	POD-1	Observation	3-Weeks
Silva et al 1992	Hernia repair	50	Supine	Bilateral causing entire tongue numb	After 24-hour postop	Observation	6 weeks
Loughman 1983	Breast surgery	150	Supine	Right side	6 days postop	Observation	6 weeks
Teichner 1971	Excision of granuloma left lung base		Supine	Right anterolateral	Postop	Observation	4 weeks

There is significant variability in the time until resolution of symptoms. Laffon et al (2001) reported a patient who resolved symptoms 2 hours post operation, while Lang and Waite (2001) reported their patient to be symptomatic 19 months after the surgery.

The majority of lingual nerve injuries were unilateral, seven reported to be left-sided, five right-sided, and the remainder not specified. However, among the 32 patients reported, 7 of them presented with bilateral lingual nerve injury and 3 presented with combined lingual nerve injury with glossopharyngeal, hypoglossal and inferior alveolar nerve injury (n = 1 for each). Furthermore, of the nine bilateral verbal nerve injuries, four of which were reported following ETT, with the five remaining being following LMA use.

### Risk factors for lingual nerve injury

Several studies/case reports have identified factors that increase the risk of lingual nerve injury during surgery.

**Type of Surgery:**

- Su et al’s (2018) retrospective case–control study highlighted that head and neck operations significantly increase the risk of postoperative lingual nerve injury.

**Operative Time:**

- Ozdamar et al (2019) demonstrated that longer operation durations were a significant risk factor for lingual nerve injury. Tessema et al’s (2006) review in micro laryngoscopy cases also linked operations lasting more than 1 hour to higher incidences of tongue paresthesia and dysgeusia, likely resulting from lingual nerve injury.

**Difficulty with Intubation:**

- Ozdamar et al’s (2019) findings showcased that experiencing difficulties during intubation was a major risk factor for lingual nerve injury.

**Age:**

- Su et al (2018) noted that younger and healthier patients had a higher risk of neuropraxia. However, Tessema et al (2006) and Ozdamar et al (2019) did not find age a significant risk factor for tongue symptoms or lingual nerve injury.

**Sex:**

- Tessema et al (2006) and Ozdamar et al (2019) studies found gender-based disparities. Female patients, according to these studies, were more prone to lingual nerve injuries post-surgery (Ozdamar et al 2019, Tessema et al 2006) Ozdamar et al (2019) specifically demonstrated a higher complication rate in females following suspension laryngoscopy.

**MP Score:**

- Ozdamar et al (2019) indicated that MP score, BMI and preoperative diagnosis were not significant predictors of lingual nerve injury.

**LMA Use:**

- Tan et al’s (2010) evaluation of LMA in 100 patients reported only one case of lingual nerve injury. However, most case reports linking LMA use to neuropraxia suggest a potential association.

This comprehensive overview of various studies provides a nuanced understanding of the multifactorial nature of lingual nerve injury, attributing it to surgery type, operative time, intubation difficulties, patient demographics (age and sex) and potential correlations with using LMA.

### Treatment of injury

In most of the case reports, expectant management was instituted. However, in five case reports, anti-inflammatory medication was used (Kim et al 2018, Lang & Waite 2001, Li et al 2022, Park & Jeong 2022, Tan et al 2010). Dexamethasone was administered at varying doses for 3 days in three of the reports (Kim et al 2018, Li et al 2022, Tan et al 2010). Park and Jeong (2022) used prednisolone for 3 days, gradually reducing the dosage by half daily. Lang and Waite (2001) also used oral steroids, but no specific details were provided. Apart from anti-inflammatory medication, Ulusoy et al (2014) and Wang et al (2021) reported the use of other vitamins, such as Vitamin E, B1 and B6 as supplements for their patients. In the Ulusoy et al’s (2014) case presentation, the patient developed anxiety and depression-like symptoms due to social isolation from their diagnosis. Patient care involved prescribing an Selective Serotonin Reuptake Inhibitor (SSRI), which was discontinued after 12 weeks.

## Discussion

Our review contributes to the existing body of evidence, highlighting an association between LMA use and lingual nerve neuropraxia. We identify significant risk factors for lingual nerve injury, emphasising female sex, extended operations, head and neck procedures, and difficulty with intubation. However, the data remain inconclusive on whether BMI, MP score and age contribute to neuropraxia.

Evidence suggests a higher risk of lingual nerve injury in females, potentially attributed to smaller jaw sizes and mouth openings (Tojyo et al 2019). Nevertheless, conflicting views on the anatomical location of lingual nerve injury exist (Mendes et al 2014). Difficulty with intubation and prolonged operations predictably increase the risk, with multiple intubation attempts causing repeated trauma and extended operations exerting more pressure on the nerve.

Our review shows that head and neck procedures are also associated with high risk for lingual nerve injury which may be due to increased airway congestion, pressure and potential airway dislodgement. Although BMI and MP scores theoretically affect airway size and intubation difficulty, limited studies warrant further investigation into their role as contributors.

We were limited in the number of studies assessing BMI and MP score as risk factors for lingual nerve injury. Theoretically, BMI and MP scores can indeed influence the airway anatomy, potentially contributing to difficulties during intubation. Larger BMI often correlates with increased adipose tissue around the neck and throat, potentially complicating airway management. Similarly, a higher MP score suggests increased oropharyngeal tissue that could obstruct the airway, making intubation more challenging. While these factors intuitively suggest a potential association with lingual nerve injury due to their impact on airway characteristics, the limited number of studies exploring this relationship does highlight a gap in the current understanding. Future investigations that delve deeper into these factors could provide valuable insights into their direct impact on lingual nerve injury during airway management

One of our review limitations is focusing solely on lingual nerve injuries related to airway management. Dental procedures and inferior alveolar blocks are indeed well recognised as common causes of lingual nerve injury. Procedures, particularly those involving wisdom tooth extraction or other oral surgeries, and inferior alveolar nerve blocks are acknowledged sources of lingual nerve injury. These procedures involve closer proximity to the lingual nerve and thus pose inherent risks. Therefore, a larger scope is necessary to better understand all forms of lingual nerve injury.

Taken together, the size of the LMA/supraglottic airway device should be chosen appropriately, generally based on patient weight. Upsizing the LMA/supraglottic airway device should be considered if cuff volume or pressure exceeds the recommended limit. Use of a cuff inflator with pressure measurement may also mitigate over-inflation. For intubation, appropriate patient positioning, attention to laryngoscopy technique and low threshold to switch to alternative airway adjuncts may avoid unnecessary pressure exerted on the soft tissue by the laryngoscopist.

In summary, it is important to note that lingual nerve injury can occur as a postoperative complication of airway management. To ensure that patients are fully informed about the risks associated with airway management, it is crucial to include lingual nerve neuropraxia as a potential complication that may arise. Anaesthetists play a vital role in communicating these risks and providing reassurance in case of complications.
